# Knowledge graph construction for heart failure using large language models with prompt engineering

**DOI:** 10.3389/fncom.2024.1389475

**Published:** 2024-07-02

**Authors:** Tianhan Xu, Yixun Gu, Mantian Xue, Renjie Gu, Bin Li, Xiang Gu

**Affiliations:** ^1^School of Information Engineering, Yangzhou University, Yangzhou, Jiangsu, China; ^2^School of Information Engineering, Yangzhou Polytechnic Institute, Yangzhou, Jiangsu, China; ^3^Department of Radiation Oncology, Yangzhou Second People's Hospital, Yangzhou, Jiangsu, China; ^4^Department of Cardiovascular, Northern Jiangsu Province People Hospital of Yangzhou University, Yangzhou, Jiangsu, China

**Keywords:** large language models, heart failure, knowledge graph, prompt engineering, TwoStepChat

## Abstract

**Introduction:**

Constructing an accurate and comprehensive knowledge graph of specific diseases is critical for practical clinical disease diagnosis and treatment, reasoning and decision support, rehabilitation, and health management. For knowledge graph construction tasks (such as named entity recognition, relation extraction), classical BERT-based methods require a large amount of training data to ensure model performance. However, real-world medical annotation data, especially disease-specific annotation samples, are very limited. In addition, existing models do not perform well in recognizing out-of-distribution entities and relations that are not seen in the training phase.

**Method:**

In this study, we present a novel and practical pipeline for constructing a heart failure knowledge graph using large language models and medical expert refinement. We apply prompt engineering to the three phases of schema design: schema design, information extraction, and knowledge completion. The best performance is achieved by designing task-specific prompt templates combined with the TwoStepChat approach.

**Results:**

Experiments on two datasets show that the TwoStepChat method outperforms the Vanillia prompt and outperforms the fine-tuned BERT-based baselines. Moreover, our method saves 65% of the time compared to manual annotation and is better suited to extract the out-of-distribution information in the real world.

## 1 Introduction

Medical knowledge graphs play an important role in clinical practice and healthcare (Abu-Salih et al., 2023). They provide data search, decision support, and visualization for diagnosis, treatment, and prognosis by integrating data from multiple sources, such as clinical guidelines, expert consensus, professional papers, and electronic health records (Xue and Zou, 2022; Wu X. et al., 2023). Among these medical knowledge graphs, the disease-specific knowledge graph constructs more targeted schemas and more comprehensive triples for specific diseases (Chandak et al., 2023). It is also more valuable in actual clinical diagnosis and treatment (Wang H. et al., 2022) and can provide mechanisms and explanations to aid in decision making (Hao et al., 2023; Yang et al., 2023).

Previous work has mainly relied on the BERT (Devlin et al., 2019) model and its variants for information extraction to build knowledge graphs. BioBERT (Lee et al., 2020) and ClinicalBert (Alsentzer et al., 2019) pre-train the text representation on biomedical text and clinical text, respectively, and perform well in medical NER and RE tasks. Gligic et al. (2019) explore the use of a combination of BERT and CRF for named entity recognition in electronic health records. (Luo et al., 2020) propose a joint learning method that combines entity recognition and relation extraction, using BERT as the basic model, and demonstrate its effectiveness on biomedical texts. Several other works (Bacanin et al., 2021, 2022; Zivkovic et al., 2022) combine machine learning and swarm intelligence methods and have shown promising results in various fields, including NLP tasks. However, the above methods have the following shortcomings: (1) To achieve good performance, these models require tens of thousands of training data, but accurately annotated medical entities and relations are scarce and time-consuming. (2) For out-of-distribution (OOD) test data (which comes from different text sources or contains new entities or relations not included in the training), model performance is low and unstable.

Recently, large language models(LLMs) have shown superior performance and emergent capabilities in a variety of natural language processing tasks. Autoregressive models such as FLamingo (Alayrac et al., 2022), LaMDA (Thoppilan et al., 2022), PaLM (Chowdhery et al., 2022), and ChatGPT (Achiam et al., 2023) etc. are able to achieve more accurate answers through techniques such as continual pre-training (Singhal et al., 2023), fine tuning (Wornow et al., 2023), and prompt engineering (Wang et al., 2024). LLMs have demonstrated competitive performance in zero-shot and few-shot settings (Brown et al., 2020), and their powerful reasoning and generalization capabilities make them well suited for dealing with out-of-distribution scenarios (Naveed et al., 2023). In addition, compared to traditional manual and model-based KG construction methods, LLMs-based KG construction methods have the following advantages: (1) LLMs are trained on a large number of natural language texts, so it is able to understand and generate natural language (Dong et al., 2019; Min et al., 2023). This gives it the ability to extract information from unstructured textual data (e.g., medical literature, electronic health records). (2) LLMs can understand complex relations between entities (Thirunavukarasu et al., 2023), allowing them to extract complex triples from text, such as “Captopril is an ACE inhibitor used to treat cardiovascular diseases such as heart failure.” (3) LLMs can generate schemas, which are templates that define entities and relations (Zhang T. et al., 2023). This is very useful for building knowledge graphs because it helps us understand and organize information.

**Figure 1:**
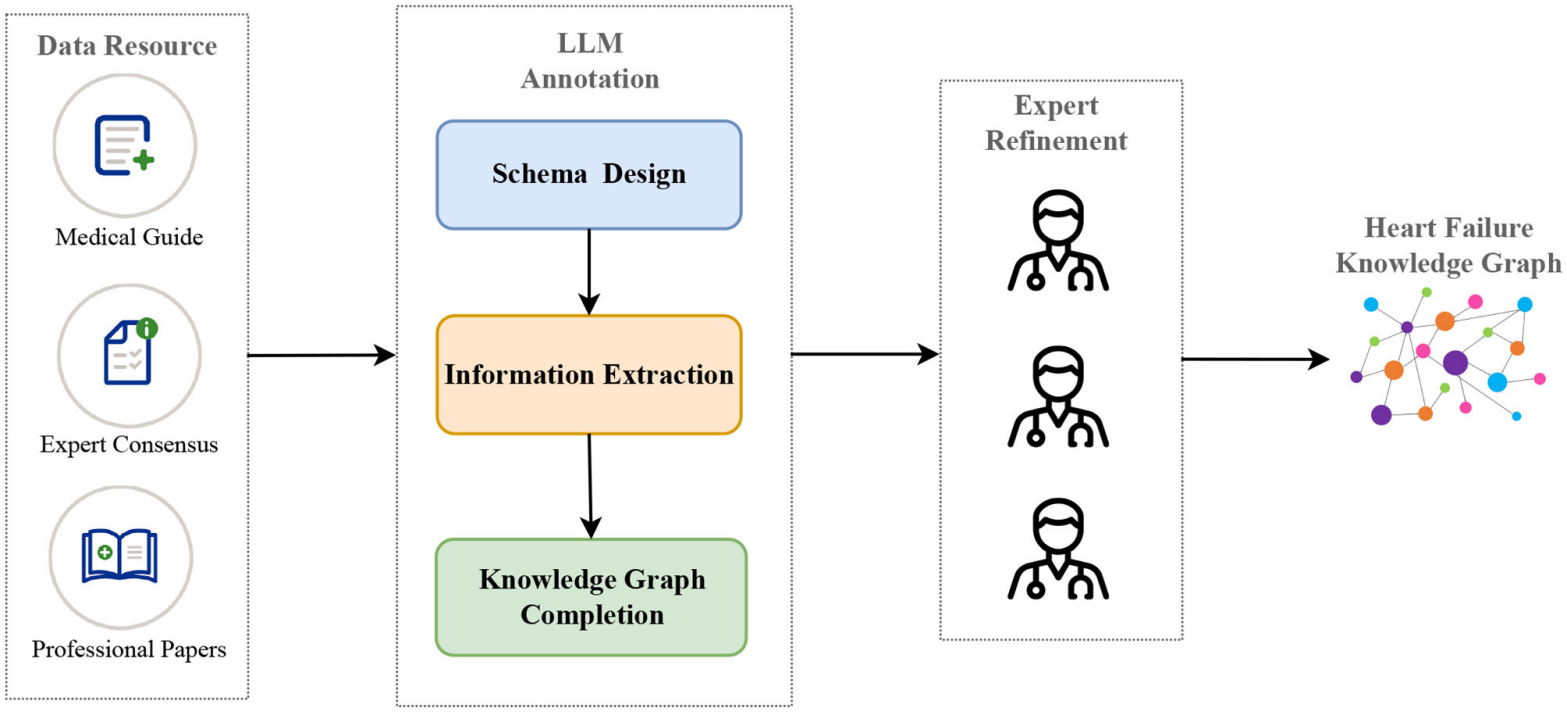
The construction pipeline of our heart failure knowledge graph (HFKG). The LLM annotation consists of three main phases: schema design, information extraction, and knowledge graph completion.

According to the above insight, in this paper, we propose a pipeline based on LLMs and prompt engineering to construct a heart failure knowledge graph to support diagnosis and treatment. Specifically, we divide the whole construction of the knowledge graph into three core phases: 1. schema design, 2. information extraction, including named entity recognition and relation extraction, 3. knowledge graph completion, including triple classification, relation prediction and link prediction. Next, three cardiovascular experts refine the entity and relation triples extracted by LLM to ensure the accuracy of the knowledge graph. Figure 1 illustrates the pipeline of our proposed method. In the information extraction phase, we maximize the potential of LLM through the TwoStepChat prompt method. In the knowledge graph completion phase, we cyclically verify the result triples of LLM in the three tasks of triple classification, relation prediction and link prediction to alleviate the hallucination of LLM. Experiments conducted on the expert-annotated gold standard heart failure dataset demonstrate that the TwoStepChat approach surpasses the performance of the Vanilla prompt. In addition, results on the public dataset show that its metrics outperform the fine-tuned BERT-based baselines. Moreover, our method reduces annotation time by 65% compared to manual annotation and is more effective in extracting out-of-distribution information in real-world scenarios. Our contributions can be summarized as follows:

- We design a pipeline to realize automatic annotation (including schema design, information extraction, and knowledge graph completion) through LLM and promt engineering, combined with expert refinement to build a specialized disease knowledge graph.
- We propose the TwoStepChat prompt to improve the performance of LLM in information extraction. Moreover, the hallucination of LLM can be effectively alleviated by our cyclic verification in knowledge graph completion.
- We construct a complete heart failure knowledge graph based on the above method. Experiments on two datasets show that the TwoStepChat method outperforms the Vanillia prompt and outperforms the fine-tuned BERT-based baselines. Compared to manual annotation, 65% of the time cost can be saved.

## 2 Related work

### 2.1 Medical knowledge graph

The main purpose of the previous medical knowledge graph construction work (Yu et al., 2017; Chandak et al., 2023; Xiong et al., 2023) is to intuitively represent the relation between medical concepts, thereby improving the user experience when retrieving medical knowledge. Shanghai Shuguang Hospital developed a traditional Chinese medicine knowledge graph (Tong et al., 2016), but faced challenges in automatically constructing recipes for clinical applications. TCMKG (Zheng et al., 2020; Yang et al., 2022) extract traditional Chinese using medicine literatures and electronic medical records for diagnosis and treatment of traditional Chinese diseases. Yuanyuan and Zhongmin (2022) summarize the progress of research and application of Chinese medical knowledge graphs. Wu T. et al. (2023) use BERT-based models to build a knowledge base for early screening and diagnosis of autism spectrum disorder. In contrast to the above work, we aim to construct a complete knowledge graph of heart failure that can support decision making for actual clinical diagnosis and treatment. In addition, our method uses LLM via prompt engineering to implement the main phases of knowledge graph construction.

### 2.2 LLMs for information extraction

LLMs perform well and have potential in information extraction (IE) tasks. Wu et al. (2024) implements structured entity extraction with LLMs. Zhou et al. (2023) uses LLMs for generalized named entity recognition, highlighting their versatility. Wei et al. (2023) propose a method using ChatGPT for zero-shot information extraction. The work (Agrawal et al., 2022; Driess et al., 2023; Singhal et al., 2023) explores the application of LLMs for medical information extraction. Our approach divides the information extraction process into three phases, named entity recognition (NER), relation extraction (RE), and entity disambiguation (ED), which effectively improves the accuracy of medical IE.

### 2.3 LLMs for knowledge graph completion

A recent comprehensive survey (Zhao et al., 2023) on the use of LLMs in knowledge graph application evaluates knowledge graph completion as a fundamental task. Two related papers (Zhu et al., 2023; Xie et al., 2023) use ChatGPT on a link prediction task in the knowledge graph and evaluate its effectiveness. Zhang Y. et al. (2023) discuss the incorporation of structural information from knowledge graphs into LLMs to achieve structural-aware reasoning. Inspired by the above work, we design three different triple completion tasks to effectively control hallucination and ensure the accuracy of LLMs through cyclic verification.

### 2.4 Chain-of-thought prompting

Chain-of-Thought (CoT) prompting, propose by Wei et al. (2022), requires LLMs to generate coherent intermediate reasoning steps leading to a final answer. As demonstrated by Kojima et al. (2022), in the few-shot scenario, LLMs reflect the CoT reasoning process. Manual CoT achieves superior performance through manually designed prompts, but recent research has focused on reducing human-intensive design efforts. Trends include decomposing complex problems into multiple sub-problems and solving them sequentially (Zhou et al., 2022) or by voting over multiple reasoning paths (Wang X. et al., 2022; Zelikman et al., 2022). Inspired by the CoT prompt, we implement NER and RE with multiple steps to improve the prediction performance of LLM.

## 3 Methodology

We use LLMs with few-shot label samples to construct a heart failure knowledge graph through three main steps: schema design, information extraction, and knowledge graph completion. This work highlights the potential of LLMs in the zero-shot or few-shot settings to significantly reduce manual annotation workload while maintaining expert-quality results.

### 3.1 Schema design

**Figure 2:**
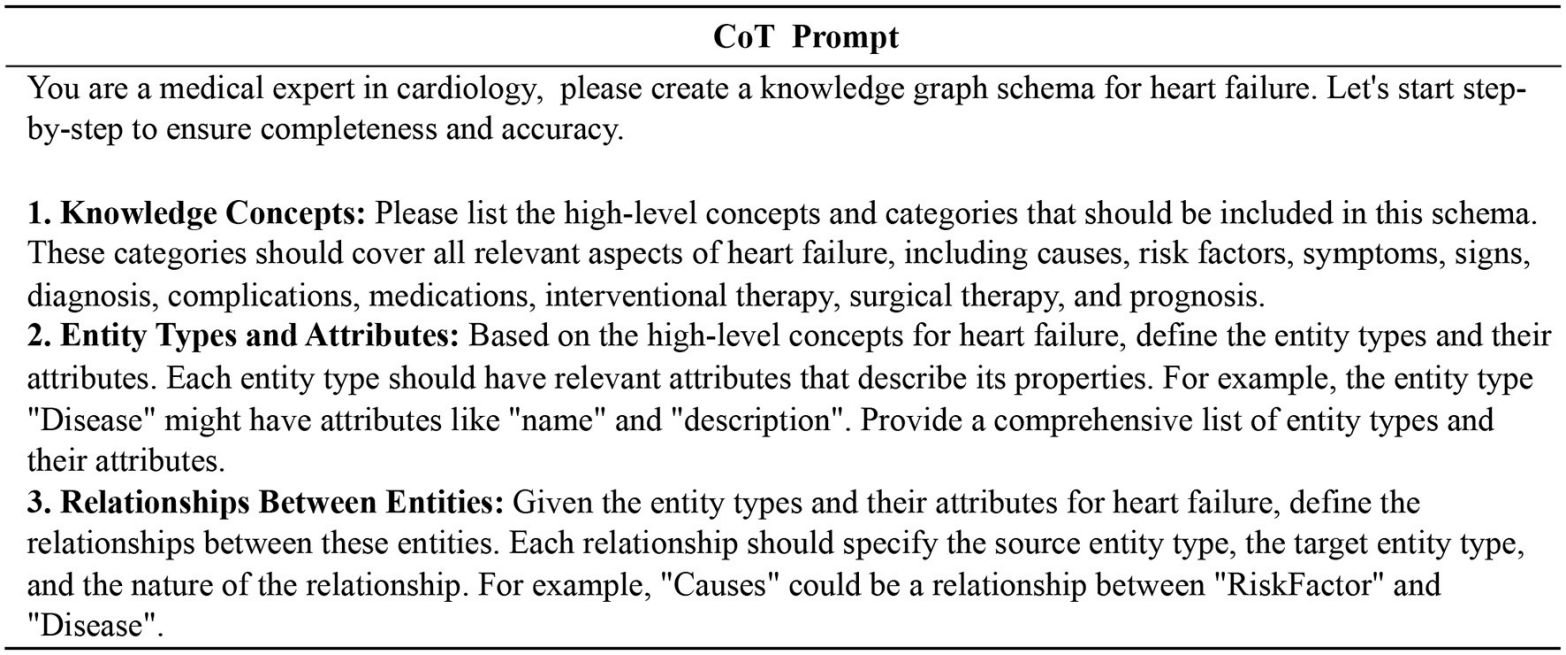
Prompt template for schema design of the heart failure knowledge graph. We get the final schema step by step through the CoT prompt.

Heart failure is a complex and comprehensive disease that can be triggered by a variety of etiologic factors and may be associated with multiple comorbidities. Its treatment includes a variety of synergistic therapeutic options such as pharmacological, interventional and surgical therapies. To construct a more fine-grained heart failure knowledge graph schema, we combine the CoT prompt (Wei et al., 2022) with the CRISPE framework (Shieh, 2023; Wang et al., 2024), and get the entity types and relation types step by step. Figure 2 illustrates our prompt template.

**Figure 3:**
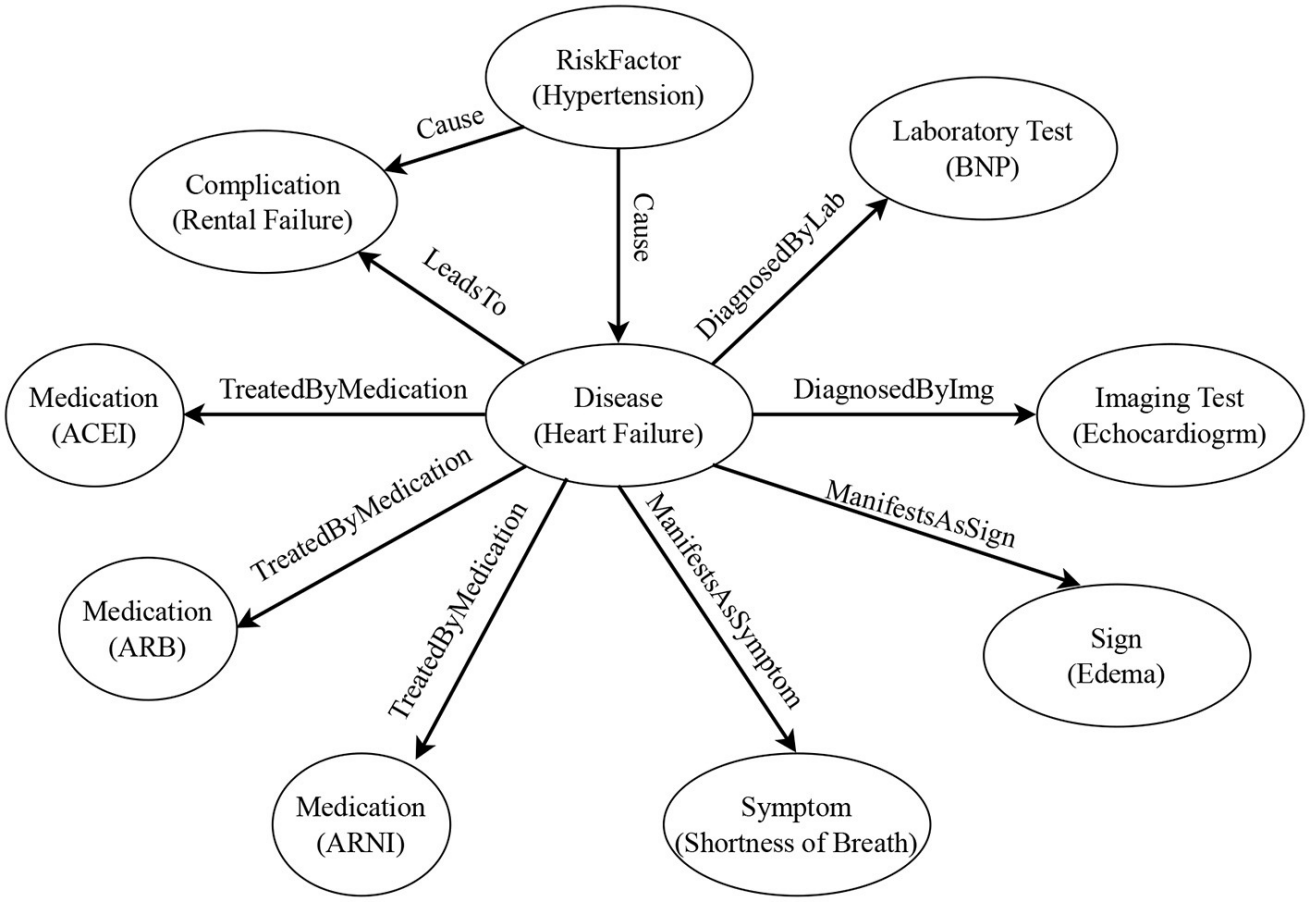
Example of the heart failure knowledge graph structure. The circles represent entity types, and the contents in parentheses are entity examples. The labels on the arrows represent relation types. The arrow points from the head entity type to the tail entity type.

In building the heart failure knowledge graph, we first define the entity schema and relation schema through LLM (see Tables 1, 2 for the resulting instances). Then, according to the schema, we automatically extract entities and relations in the document through LLM, and fill the knowledge graph with specific instance data. Figure 3 shows an example of the structure of our knowledge graph.

**Table 1:** Part of the entity schema generated by the LLM (ChatGPT3.5).

**Entity type**	**Attribute name**	**Attribute type**	**Description**
Disease	Name	String	Name of the disease
	Description	String	Description of the disease
Cause	Name	String	Name of the cause
	Description	String	Description of the cause
RiskFactor	Name	String	Name of the risk factor
	Description	String	Description of the risk factor
Symptom	Name	String	Name of the symptom
	Description	String	Description of the symptom
Sign	Name	String	Name of the sign
	Description	String	Description of the sign
LaboratoryTest	Name	String	Name of the laboratory test
	Description	String	Description of the laboratory test
ImagingTest	Name	String	Name of the imaging test
	Description	String	Description of the imaging test
Complication	Name	String	Name of the complication
	Description	String	Description of the complication
Medication	Name	String	Name of the medication
	Description	String	Description of the medication
	Dosage	String	Dosage of the medication
InterventionalTherapy	Name	String	Name of the interventional therapy
	Description	String	Description of the interventional therapy
SurgicalTherapy	Name	sTring	Name of the surgical therapy
	Description	String	Description of the surgical therapy
Prognosis	Name	String	Name of the prognosis
	Description	String	Description of the prognosis

**Table 2:** Part of the relation schema generated by the LLM (ChatGPT3.5).

**Relation type**	**Source entity type**	**Target entity type**	**Description**
Cause	RiskFactor	Disease	Risk factor causes disease
ManifestsAsSymptom	Disease	Symptom	Disease manifests as symptom
ManifestsAsSign	Disease	Sign	Disease manifests as sign
DiagnosedByLab	Disease	LaboratoryTest	Disease diagnosed by laboratory test
DiagnosedByImg	Disease	ImagingTest	Disease diagnosed by imaging test
LeadsTo	Disease	Complication	Disease leads to complication
TreatedByMedication	Disease	Medication	Disease treated by medication
TreatedByIntervention	Disease	InterventionalTherapy	Disease treated by interventional therapy
TreatedBySurgery	Disease	SurgicalTherapy	Disease treated by surgical therapy
PrognosisOfDisease	Disease	Prognosis	Prognosis of disease

### 3.2 Information extraction

**Figure 4:**
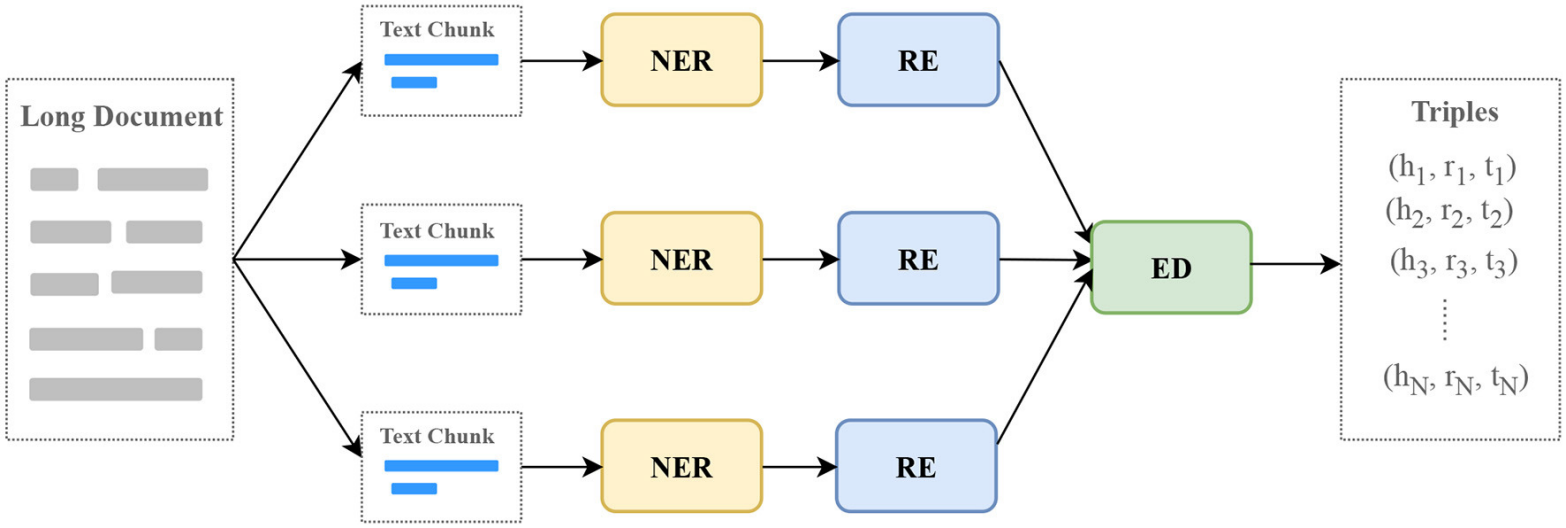
The process of extracting information with LLMs. NER, named entity recognition; RE, relation extraction; ED, entity disambiguation.

Medical guidelines, expert consensus, and professional papers are long documents. We break these documents into text chunks based on paragraph breaks, end of sentence markers, and line breaks to ensure that each text chunk is within the maximum input length of the model. Then, the text chunks are used as input and go through three processes of named entity recognition (NER), relation extraction (RE), and entity disambiguation (ED) to obtain output triples, as shown in Figure 4.

We decompose the NER task and the RE task into two steps to improve the accuracy of the LLM response, which we call **TwoStepChat**. Each step consists of one or more rounds of conversation with the LLM. In the first step, our goal is to find out the existing entity types and relation types in the NER and RE tasks, respectively. In the second step, we further extract the entities in the NER task and the (head entity, relation, tail entity) triples in the RE task based on the types extracted in the first step using appropriate task-specific prompt templates.

#### 3.2.1 Named entity recognition

**Figure 5:**
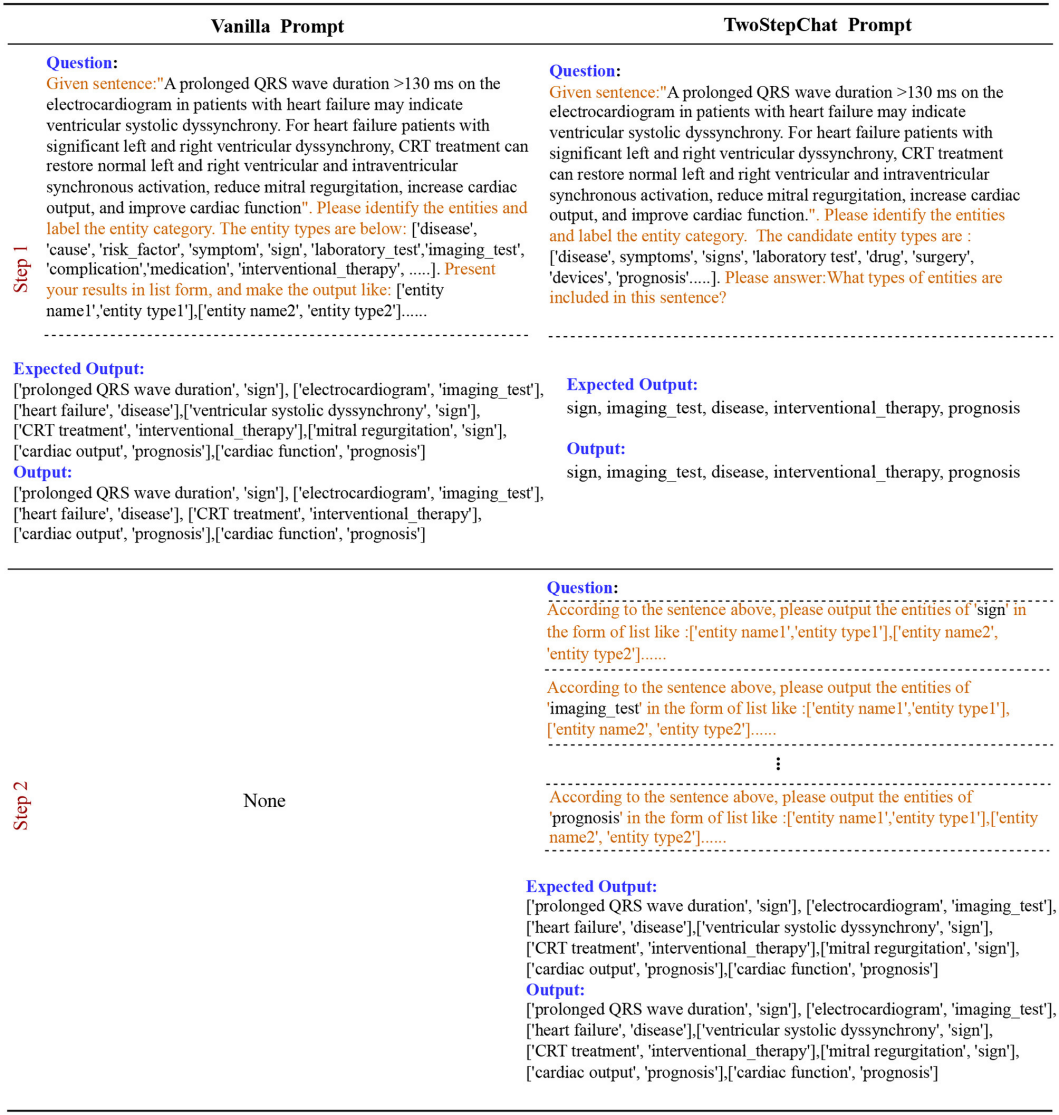
Description of the Vanilla prompt vs. our TwoStepChat prompt in the NER task. The text highlighted in orange represents the prompt template.

For the NER task, the first step is to determine the entity type contained in the text chunk, given a list of all entity types. In the second step, the goal of each round is to extract one entity type. The total number of rounds in the second step is equal to the number of entity types contained in the text chunk obtained in the first step. If no entity type is obtained in the first step, the second step is skipped. We do not use BIO annotations because it is difficult for autoregressive language models in a zero-shot setting. See Figure 5 for an example of our method with respect to NER.

#### 3.2.2 Relation extraction

**Figure 6:**
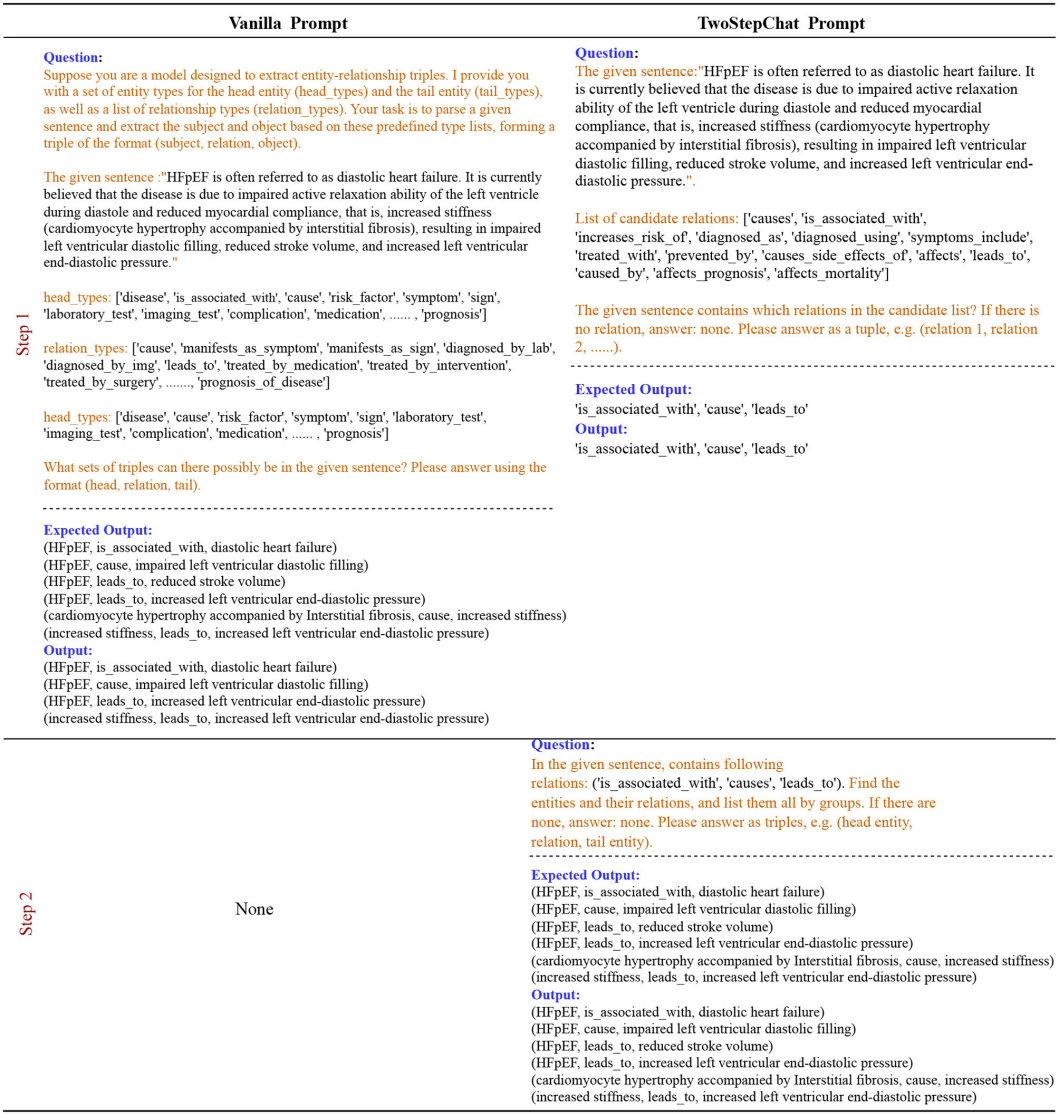
Description of the Vanilla prompt vs. our TwoStepChat prompt in the RE task. The text highlighted in orange represents the prompt template.

We define the input text chunk as *x*, the question prompt as *q*. The RE task is to predict triples *T* = {(*h*_1_, *r*_1_, *t*_1_), ⋯  , (*h*_*n*_, *r*_*n*_, *t*_*n*_)}, where *n* donates the number of triples, *type*((*h*_*i*_, *r*_*i*_, *t*_*i*_)) ∈ *R* and *R* is the set of all the relation types. The two steps of RE process can be formulated in Equation 1.


(1) $$\begin{array}{l} p((h,r,t)|x,q)=\underset{step\;1}{\underset{︸}{p(r|x,q_{1})}}\underset{step\;2}{\underset{︸}{p((h,t)|q_{r})}} \end{array}$$

where *q*_1_ is the question generated in step 1 using all the relations *R* to fill the template of LLM and get the relation types *r* existing in the text. *q*_*r*_ is a question generated in step 2 using the corresponding template based on the existing types *r* in step 1 to generate triples. We omitted *x* in step 2 because ChatGPT can automatically maintain the session for each round of QA. See Figure 6 for an example of our method with respect to RE.

#### 3.2.3 Entity disambiguation

When building a knowledge graph, triples from different documents inevitably have entity ambiguity problems. We design prompt templates and interact with LLM to guide it to perform entity disambiguation based on entity-related triples. For example, in medicine, “Heart Failure” and “Congestive Heart Failure” are often considered the same entity because they both refer to a condition in which the heart is unable to pump blood effectively. “Atrial Fibrillation” and “Ventricular Fibrillation” are different entities, they are arrhythmias that occur in different parts of the heart and have different clinical characteristics and consequences.

We first compute the Jaccard similarity of all head entities based on the mined triples to filter out candidate entity pairs for disambiguation. Given two entities *A* and *B* with relation sets *R*_*A*_ and *R*_*B*_, and tail entity sets *T*_*A*_ and *T*_*B*_, the Jaccard similarity *J*(*A, B*) is computed in Equation 2.


(2) $$\begin{array}{l} J\left(A,B\right)=\frac{\left|R_{A}\cap R_{B}\cap T_{A}\cap T_{B}\right|}{\left|R_{A}\cup R_{B}\cup T_{A}\cup T_{B}\right|} \end{array}$$

where |*R*_*A*_ ∩ *R*_*B*_ ∩ *T*_*A*_ ∩ *T*_*B*_| denotes the cardinality of the intersection of relations and tail entities between entities *A* and *B*. |*R*_*A*_ ∪ *R*_*B*_ ∪ *T*_*A*_ ∪ *T*_*B*_| represents the cardinality of the union of all relations and tail entities associated with entities *A* and *B*. The Jaccard similarity measure *J*(*A, B*) quantifies the degree of similarity between entities *A* and *B* based on their shared relations and tail entities.

**Figure 7:**
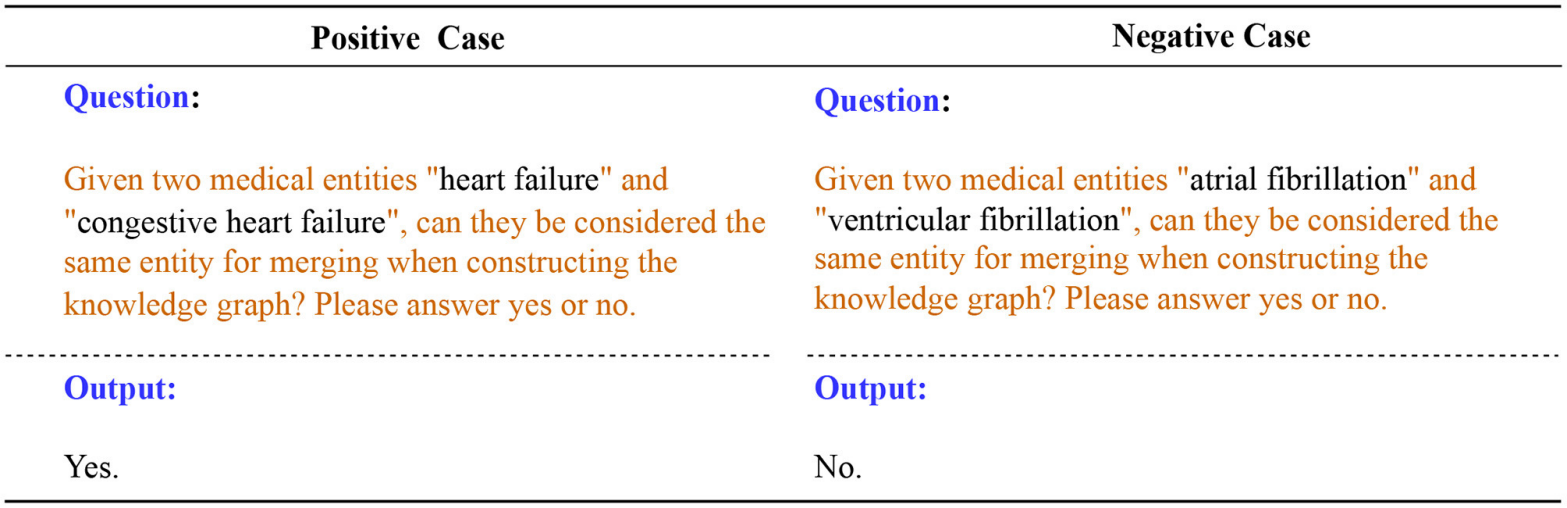
Positive and negative cases for entity disambiguation using LLM. The text highlighted in orange represents the prompt template.

Next, we fill in the candidate entity pairs in the prompt template as input to LLM for entity disambiguation. Figure 7 shows two cases, one positive and one negative. LLM performs reasoning and interpretation based on the information provided, helping us to disambiguate entities and provide merged results. Merging and unifying duplicate entities ensures that entities in the knowledge graph are unique and improves the accuracy and consistency of the knowledge graph.

### 3.3 Knowledge graph completion

In this subsection, we discuss how to complete the heart failure knowledge graph with the above triples mined by the LLM. We implement triple completion through the following three tasks: triple classification, relation prediction, and link prediction. For each of the three tasks, we design different prompts for the LLM.

#### 3.3.1 Triple classification

Given a triple (head entity, relation, tail entity), the binary classification task is aim to classify the triple as true or false. For example, given (hypertension, increases_risk_of, heart failure), the prompt template for LLMs is as follows: “Based on the medical knowledge of cardiovascular specialists, hypertension increases risk of heart failure? Please answer true or false.” and the desired output for LLMs is “True”.

#### 3.3.2 Relation prediction

Given a head entity and a tail entity, the task is to predict the relation between them. For example, given the head entity “hypertension” and the tail entity “heart failure”, the task is to predict whether their relation is “caused”. We design the following prompt template: “What is the medical relation between hypertension and heart failure? Please select the best answer based on your medical expertise from the following option list: [‘causes', ‘is_associated_with', ‘increases_risk_of', ‘diagnosed_as', ‘diagnosed_using', ‘symptoms_include', ‘treated_with', ‘prevented_by', ‘causes_side_effects_of', ‘affects', ‘leads_to', ‘caused_by', ‘affects_prognosis', ‘affects_mortality'].” The expected answer is “increases_risk_of”.

#### 3.3.3 Link prediction

Given a head entity and a relation, the goal of the task is to predict the tail entity related to the head entity. Given a tail entity and a relations, the task is to predict the head entity. For example, given the head entity “hypertension” and the relation “increases_risk_of”, the task is to predict the tail entity “heart failure”. We define the following prompt templates for LLMs: “Hypertension increases risk of what disease?” is used to ask the tail entity, “What disease increases risk of heart failure?” is used to ask the head entity.

**Figure 8:**
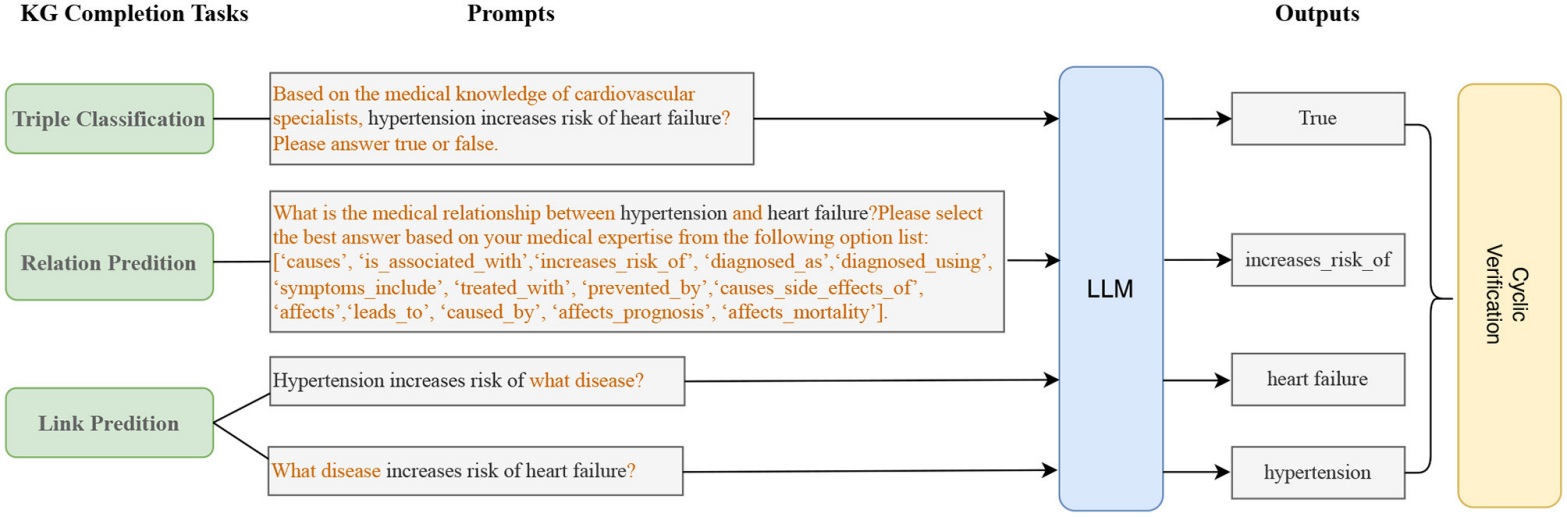
Knowledge graph completion (KGC) with LLMs. We ensure the accuracy and professionalism of knowledge graph completion through cyclic verification of three tasks.

The three tasks can complement and confirm each other, which we call **triple cyclic verification**. For example, we can use the triple classification task to verify that the results of the relation prediction task are correct; we can also use the relation prediction task to verify the results of the link prediction; the two methods of link prediction can also confirm each other, as shown in Figure 8. We use **triple cyclic verification** to try to avoid the hallucinations(Ye et al., 2023) of LLMs.

### 3.4 Expert refinement

To build a medical knowledge graph, especially a disease-specific knowledge graph, manual annotation is essential. Manual annotation requires medical expertise and professional training, and the process is time-consuming and expensive. Because annotation typically involves marking text areas in long documents, it requires a high level of concentration on the part of human annotators to avoid errors. As a result, annotators are prone to fatigue. However, relying on model predictions alone cannot guarantee the accuracy of the results, which is critical for disease-specific knowledge graphs.

Based on the above considerations, we first use LLM to quickly design the schema and extract the entities and relations of the knowledge graph through prompt engineering. Each part of the knowledge graph is then manually verified by experts, which we call **“expert refinement”**, as shown in Figure 1. We believe that verifying and supplementing the triples extracted by the model saves more manpower, time, and money than relying entirely on manually annotating triples from scratch. Our human team consists of 10 members, each with a background in cardiovascular medicine and experience in medical NLP annotation. The team of 10 is divided into two groups. The first group consists of medical residents or graduate students specializing in cardiovascular medicine and is called the **“annotation group”**. They are responsible for collecting important heart failure guidelines, expert consensus and professional papers and manually annotating the entities and relations in them to serve as a control group in the experiment for comparison with the extraction results of LLM. The second group consists of three cardiovascular directors and medical experts and is called the **“refinement group”**. Their tasks include schema revision and quality control, evaluation of the entities and relations marked by LLM and the “annotation group”, correction of incorrect annotations, and completion of missing annotations.

## 4 Experiment

### 4.1 Datasets and base LLM

#### 4.1.1 BioRED dataset

BioRED (Luo et al., 2022) is a widely used public dataset for entity and relation extraction. The dataset contains multiple entity types (e.g., gene/protein, disease, chemistry) and relationship pairs (e.g., gene-disease; chemistry-chemistry) at the document level. In addition, BioRED annotates whether each relation describes a new discovery or known background knowledge, allowing automatic extraction algorithms to distinguish between novel and background information. The dataset merges similar relation types to reduce management complexity while increasing the number of instances of each relation type. The BioMED dataset annotates 600 PubMed National Center for Biotechnology Information (2024) abstracts, including 400 for the training sets, 100 for the development sets, and 100 for the test sets. The dataset contains 4 types of entities, namely disease (D), gene (G), chemical (C), and variant (V). In terms of relationships, it contains eight biologically meaningful non-directional relationship types, such as positive and negative correlations, which are used to characterize the relationship between pairs of entities.

#### 4.1.2 HF dataset

The HF dataset is a private dataset of heart failure entities and relations that we have constructed. We divide the collected heart failure document data from guidelines, expert consensus, professional articles, and medical websites into text chunks, with each chunk containing 500–700 words. We end up with a total of 287 text chunks. The dataset is annotated by three cardiovascular experts from the refinement group, and an incremental evaluation is used to ensure the authority of the annotation results. 187 text chunks in the dataset are used for the training set, 50 for the development set and 50 for the test set. The HF dataset contains 12 types of entities and 10 types of relations, as shown in Tables 1, 2.

#### 4.1.3 Base LLM

We use ChatGPT3.5 (OpenAI, 2023) as the base LLM for automatic annotation in the following experiments. The GPT-3.5-turbo-16k API is chosen, it extends the token limit to 16,000 tokens and is useful for handling longer contexts and allows us to test more few-shot samples.

### 4.2 Expert annotation

All the three experts in “refinement group” have extensive clinical and academic research experience in cardiovascular medicine. Among them, Expert A is the Director of the Department of Cardiovascular Medicine in a tertiary hospital with thirty years of clinical and scientific research experience; Expert B is the Director of Cardiovascular Surgery with rich cardiovascular clinical experience and bioinformatics research background; Expert C is the Deputy Director of the Department of Cardiovascular Medicine with rich cardiovascular clinical experience and very familiar with knowledge graph and artificial intelligence.

We adopt an incremental evaluation method, that is, for each triplet, if two of the three experts give the same result, the result is taken as the ground truth. This method can effectively reduce the bias of a single evaluator and improve the reliability of the evaluation results. To evaluate the consistency of the experts' annotation results, we calculate the standard deviation and Cohen's kappa coefficient of the three experts. The results show that the three experts' evaluation of the triplet has a high degree of consistency, with a standard deviation of 0.34 and a Cohen's Kappa coefficient (Cohen, 1960) of 0.85, indicating that the evaluation results among the experts have high reliability.

### 4.3 Model comparison

#### 4.3.1 Model performance on the HF dataset

First, we compare the performance of our TwoStepChat prompt to the vanilla prompt on the HF dataset. Table 3 shows the result metrics under the zero-shot and few-shot settings. Under the zero-shot setting, TwoStepChat's F1 score increases by 1.5% compared to vanilla. Under the few-shot setting, we provide 6, 10, and 20 shot examples, respectively. The number of positive and negative examples is the same, and all shot examples are taken from the gold standard annotated by the three experts. Using the TwoStepChat prompt, which provides 20 shot examples, the F1 score is 4% higher than zero-shot. Overall, the F1 score of TwoStepChat is higher than that of Vanilla, and the F1 score of few-shot is higher than that of zero-shot. This further confirms the rationality of our TwoStepChat design, and also shows that adding more golden examples to the prompt context can effectively improve the performance of LLM.

**Table 3:** Performance comparison between our TwoStepChat prompt and the Vanilla prompt on the HF dataset.

**Model**		**NER**			**RE**		
	**Shots**	**Precision**	**Recall**	**F1**	**Precision**	**Recall**	**F1**
Vanilla-zeroshot	0	80.05	88.00	83.83	74.67	80.78	77.61
TwoStepChat-zeroshot	0	82.33	88.50	85.31	78.26	84.50	81.27
Vanilla-fewshot	6	80.87	90.00	85.18	75.50	82.32	78.77
Vanilla-fewshot	10	87.58	86.75	87.16	79.25	82.60	80.89
Vanilla-fewshot	20	88.45	**91.25**	89.83	80.80	**85.10**	82.90
TwoStepChat-fewshot	6	85.94	89.10	87.49	78.35	82.52	80.38
TwoStepChat-fewshot	10	87.35	91.35	89.31	80.68	84.33	82.47
TwoStepChat-fewshot	20	**88.59**	90.20	**89.39**	**82.72**	83.75	**83.23**

Bold text indicates the highest score.

#### 4.3.2 Model performance on the BioRED dataset

To further verify the feasibility of our proposed method, we compare ChatGPT3.5 based on TwoStepChat prompts and the fine-tuned BERT-based baselines on the public BioRED dataset. We choose BERT-GT and BiomedBERT as our baseline models. BERT-GT (Lai and Lu, 2020) is an improved BERT model that combines the bidirectional encoder representation of the transformer and the graph transformer. BERT-GT is applicable to other biomedical relation extraction tasks. BiomedBERT (Gu et al., 2021) is a pre-trained BERT model specifically for the biomedical domain. It uses abstracts and full-text articles from PubMed and PubMedCentral for training and performs well in biomedical entity recognition and relation extraction tasks.

From the experimental results in Table 4, it can be seen that our TwoStepChat method performs significantly better than other baseline models in both tasks. Specifically, in the NER task, the TwoStepChat method achieved an accuracy of 83.50%, a recall rate of 80.45%, and an F1 value of 81.96%, which is nearly 22% higher than the F1 value of 67.09% in the BERT model. Compared to BiomedBERT, although the latter has achieved relatively good performance in the biomedical field, TwoStepChat still has an F1 value nearly 6 percentage points higher. This fully demonstrates the accuracy and robustness of TwoStepChat in entity recognition. In the RE task, TwoStepChat also performed excellently, achieving accuracy, recall and F1 values of 68.25, 67.67, and 67.96%, respectively. Compared to the F1 value of 52.78% in the BERT model, the improvement was more than 25%. Compared to BiomedBERT, TwoStepChat also increased its F1 score by almost 9 percentage points. This significant performance improvement demonstrates the effectiveness of TwoStepChat in relation extraction tasks.

**Table 4:** Performance comparison of our method and baseline models on the BioRED dataset.

**Model**	**NER**			**RE**		
	**Precision**	**Recall**	**F1**	**Precision**	**Recall**	**F1**
BERT (Devlin et al., 2019)	70.57	68.82	67.09	54.03	51.58	52.78
BERT-GT (Lai and Lu, 2020)	75.38	73.04	72.15	56.70	56.60	56.57
BiomedBERT (Gu et al., 2021)	76.64	73.58	75.07	60.38	57.58	58.93
TwoStepChat (ours)	83.50	80.45	81.96	68.25	67.67	67.96

### 4.4 Evaluation of ED and KGC

The performance of entity disambiguation and knowledge graph completion on the HF dataset can be seen in Table 5. The role of entity disambiguation in our graph construction process is to maintain the consistency of entities in the knowledge graph. Through extensive evaluation by three experts, the precision of ED on our HF dataset is 92.75%, and the recall can reach 88.60%, reflecting the value of LLM in entity disambiguation, especially in identifying aliases and abbreviations.

**Table 5:** Performance of entity disambiguation and knowledge graph completion on the HF dataset.

**Model**	**Precision**	**Recall**	**F1**
Entity disambiguation	92.75	88.60	90.44
Cyclic verification	95.33	85.72	90.25
Triple classification	90.15	91.37	90.75
Relation prediction	88.67	88.81	88.74
Link prediction	86.58	87.74	87.05

For knowledge graph completion, the precision of our cyclic verification is 95.33%. This can reflect that the cyclic verification can effectively reduce the hallucination of LLM. Through knowledge graph completion, we can mine potential triples through the reasoning ability of LLM, which can improve the efficiency of knowledge graph construction on the open data.

### 4.5 Quality evaluation

In this subsection, we will compare the manual annotation results from “annotation group” and the automatic annotation results from ChatGPT3.5. Inspired by the work (Uzuner, 2009), we adopt a phrase-level evaluation method to evaluate the quality of the model. At the token level, each token in the text is counted individually, while at the phrase level, they are counted as a whole. For example, [“100”, “mg”] and “100 mg” represent token-level and phrase-level entities, respectively. Extracted entities are evaluated in the NER task, while the RE task evaluates both entities and relations. We choose ChatGPT3.5 with TwoStepChat-fewshot-10 prompt as the LLM model. During manual annotation, all text chunks are evenly distributed among the seven members of the “annotation group”.

The results can be seen in Table 6, the precision of Munal Annotation is slightly higher than that of LLM Annotation. However, LLM Annotation achieves higher recall rates and F1 scores in both NER and RE task, which is very important for knowledge graph construction. This result shows that LLM can match or even outperform human annotators with only a few shots of 10 samples. Further analysis shows that neither LLM Annotation nor Manual Annotation is accurate enough compared to the gold standard (ground truth), reflecting the importance of expert-level refinement.

**Table 6:** Performance comparison between LLM annotation and manual annotation.

**Model**	**NER**			**RE**		
	**Precision**	**Recall**	**F1**	**Precision**	**Recall**	**F1**
LLM Annotation	87.35	91.35	89.30	80.68	84.33	82.46
Manual Annotation	88.70	88.16	88.43	81.24	80.67	80.95

The scores in the table use the golden annotations of the expert group as ground truth and are calculated from the extracted entities and relationships from all 287 text chunks.

### 4.6 Efficiency evaluation

To quantify the time cost savings of our pipeline, we separately count the time for manual annotation and expert refinement as well as the time for LLM annotation and expert refinement on all 287 text chunks and plot them as box plots.

**Figure 9:**
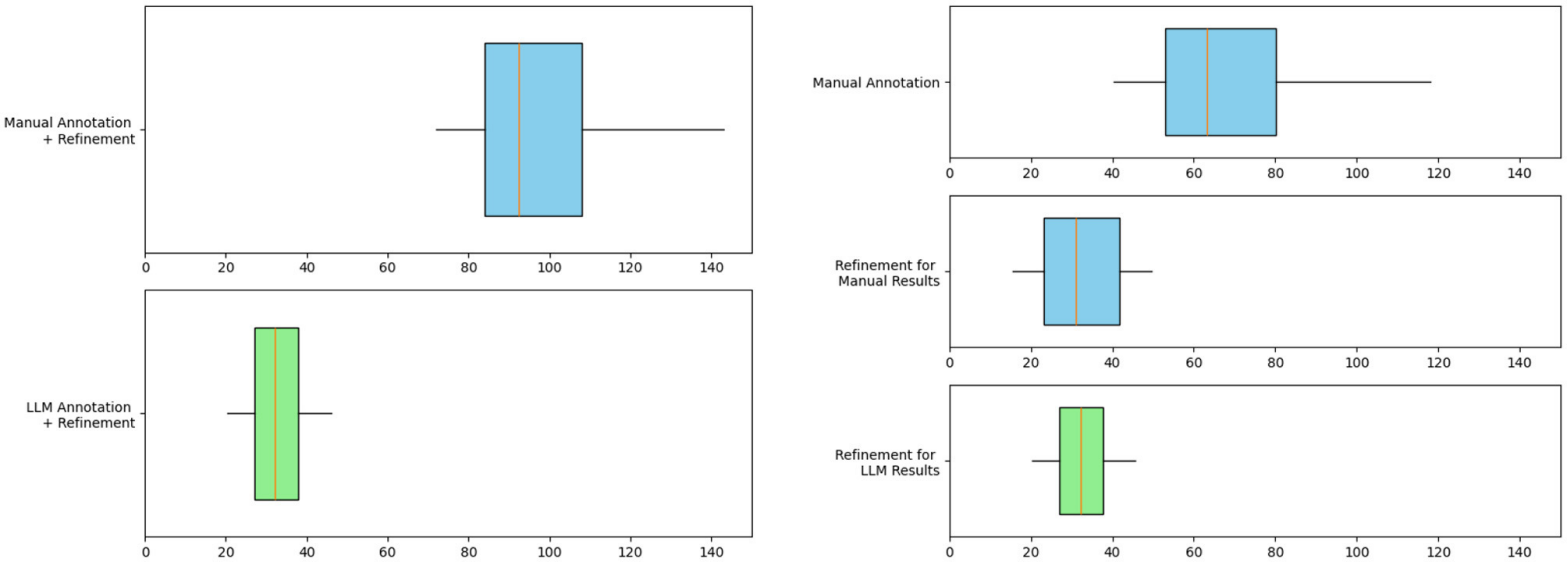
Box plot evaluating the efficiency of knowledge graph construction for heart failure. The graph on the left compares the total time spent on manual annotation + refinement and LLM annotation + refinement. The figure on the right shows the detailed time cost of each step. The blue box represents manual work and the green box represents LLM work.

Results in Figure 9 shows that the integration of LLM leads to a significant reduction in the time cost of knowledge extraction from the knowledge graph. The horizontal axis in the figure represents time in minutes, counting the time it takes different methods to extract heart failure-related medical entities and relations from a text chunk containing 500–700 words. The average time to generate the final knowledge graph triplet from text chunks using LLM is reduced by 65% from 92.6 to 32.1 min. The right subgraph provides a detailed view of the time cost of the annotation and refinement phases. Due to the need to annotate from scratch, Manual Annotation has the highest time cost with an average time of 63.3 min per text chunk. Since the time of LLM Annotation on a single chunk of text is very short, about 1 minute, we ignore this time cost. The time cost for refinements after manual annotation and LLM annotation is 30.7 and 32.1 min per text chunk, respectively. These results reflect the efficiency benefits of LLM automated annotation.

### 4.7 Knowledge graph visualization

**Figure 10:**
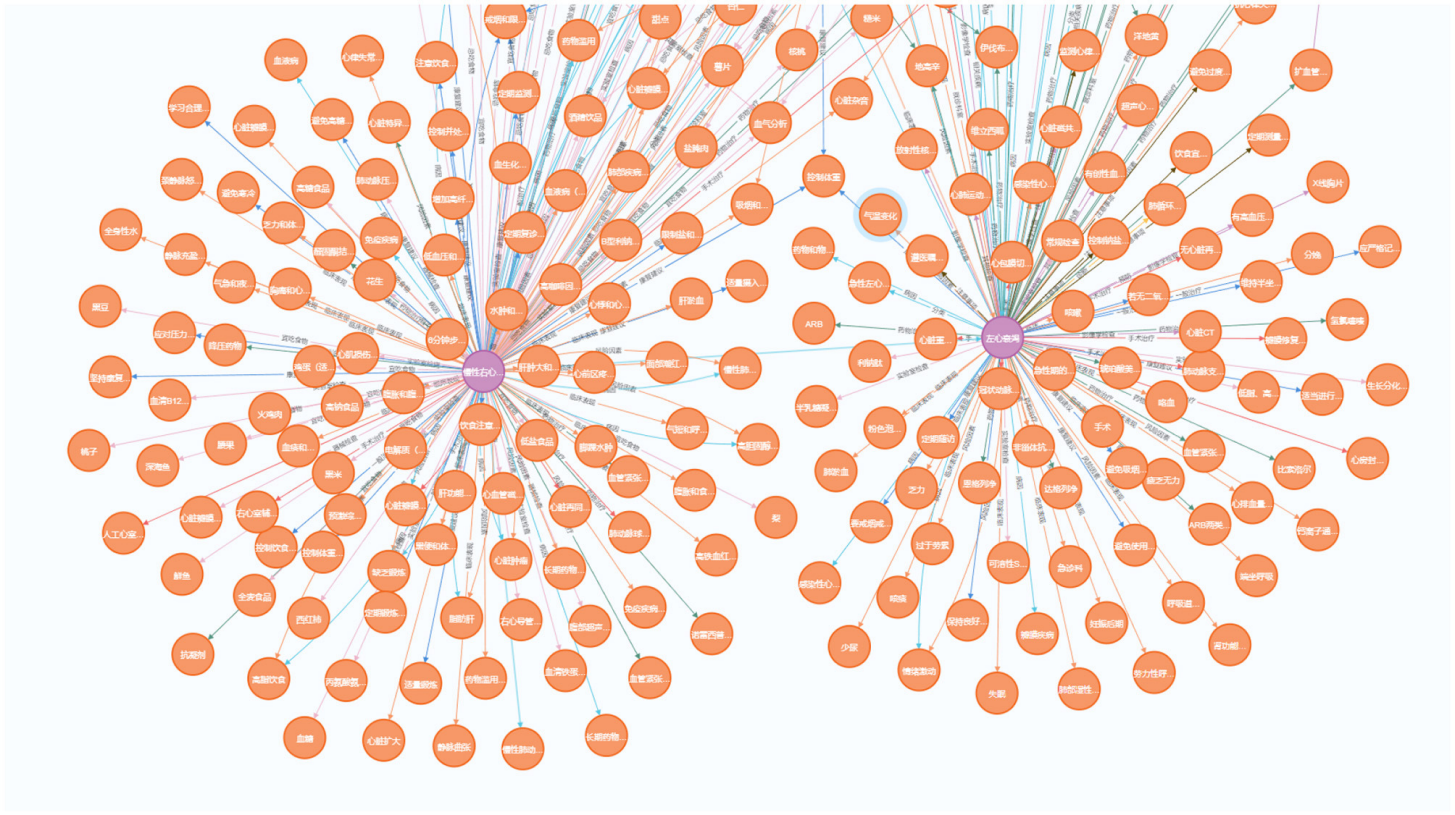
Demonstration of a sub-graph in our heart failure knowledge graph (HFKG). To facilitate the use of Chinese scenes, we translate the extracted triples into Chinese and verify them.

Since heart failure may caused by various diseases and often has other comorbidities, our knowledge graph focus on heart failure and expand to other diseases. These diseases include common cardiovascular diseases such as hypertension, atrial fibrillation, arrhythmias, and coronary artery disease. We use Neo4j software to store and visualize our Heart Failure Knowledge Graph (HFKG). The HFKG contains a total of 1,258 entities and 10,734 triples. Table 7 shows the statistics for different types of triples, respectively. To facilitate the use of Chinese scenes, we translate the extracted triples from English to Chinese using Google Translate. Figure 10 shows a subgraph instance of HFKG. The above data visualizes the diversity and richness of knowledge related to the diagnosis, treatment, and prognosis of heart failure in our knowledge graph.

**Table 7:** Heart failure knowledge graph entity type statistics and related triplet statistics.

**Type**	**Number of entities**	**Number of triples**
Disease	152	1,210
Cause	98	1,524
RiskFactor	128	1,810
Symptom	204	2,006
Sign	86	510
LaboratoryTest	159	806
ImagingTest	105	508
Complication	52	404
Medication	150	708
InterventionalTherapy	74	456
SurgicalTherapy	40	288
Prognosis	54	612

### 4.8 Question and answering application

We curate a professional Q&A dataset from medical experts containing 100 clinical questions related to diagnosis, treatment, and prognosis of heart failure, including simple queries and multi-hop queries. Using this dataset as a benchmark, we compare our Chinese heart failure knowledge graph with its open source counterparts. For this purpose, we construct a simple KBQA system to query the knowledge graphs and respond via templates. Compared with the following open source counterparts, our HFKG is able to respond more professionally and can handle a variety of complex clinical queries related to heart failure, as shown in Table 8.

**Table 8:** Example of instance comparison between our HFKG Q&A system and open source counterparts.

Question 1:	**Treatment drugs for right heart failure?**
Ling-Yi (Xia et al., 2022)	Laboratory tests for right heart failure include electrocardiography, cardiac color Doppler ultrasound, etc. The main diagnosis of heart failure relies on clinical data, signs, and impaired function.
MedicalKG (Liu, 2024)	I'm sorry, no relevant information found.
**Ours**	Treatment drugs for right heart failure: digoxin, dopamine, isosorbide dinitrate, nitroglycerin, sodium aescinate, etc.
Question 2:	**What are the surgical treatments for heart failure?**
Ling-Yi (Xia et al., 2022)	Surgical treatments for heart failure include cardiac resynchronization therapy and heart transplantation.
MedicalKG (Liu, 2024)	I'm sorry, no relevant information found.
**Ours**	Surgical treatments for heart failure: valve repair or replacement surgery, coronary artery bypass grafting, cardiac assist devices, radiofrequency ablation, coronary artery bypass surgery, heart transplantation, implantable cardioverter-defibrillator.

Ling-Yi (Xia et al., 2022): A question-answering system based on a Chinese Medical Knowledge Graph (CMKG) and a large Chinese Medical Conversational Question-Answering (CMCQA) dataset.

MedicalKG (Liu, 2024): A question-answering system built on a disease-centered knowledge graph in the medical field.

## 5 Conclusion

In this paper, we use LLM and prompt engineering to quickly build a heart failure knowledge graph to provide decision support for actual medical diagnosis and treatment. We design a novel pipeline to realize automatic annotation of medical entities and relations through LLM, and to ensure the accuracy of the knowledge graph through expert refinement. Experiments on two datasets show that the TwoStepChat method outperforms the Vanillia prompt and outperforms the fine-tuned BERT-based baselines. Moreover, our pipeline can save 65% of the time cost compared to manual annotation from scratch.

Our main goal is to build and demonstrate a complete process pipeline, and in the experiment we only extract medical triples based on ChatGPT3.5, which is a practical limitation. In future work, we will explore the use of professional medical LLMs or fine-tune the base LLM on medical corpus to further improve the model's performance on NER, RE, and knowledge graph completion tasks.

## Data availability statement

The raw data supporting the conclusions of this article will be made available by the authors, without undue reservation.

## Author contributions

TX: Conceptualization, Formal analysis, Investigation, Methodology, Software, Visualization, Writing – original draft, Writing – review & editing. YG: Data curation, Formal analysis, Resources, Validation, Writing – original draft, Writing – review & editing. MX: Formal analysis, Methodology, Software, Validation, Visualization, Writing – review & editing. RG: Data curation, Resources, Validation, Writing – review & editing. BL: Funding acquisition, Resources, Supervision, Validation, Writing – review & editing, Conceptualization, Investigation. XG: Resources, Supervision, Writing – review & editing.
